# The international PROGRESS registry of patients with severe sepsis: drotrecogin alfa (activated) use and patient outcomes

**DOI:** 10.1186/cc7936

**Published:** 2009-06-30

**Authors:** Greg Martin, Frank M Brunkhorst, Jonathan M Janes, Konrad Reinhart, David P Sundin, Kassandra Garnett, Richard Beale

**Affiliations:** 1 Division of Pulmonary, Allergy, and Critical Care, Emory University, 615 Michael Street, Atlanta, Georgia, 30322, USA; 2 Department of Anesthesiology and Intensive Care, Friedrich Schiller University, Erlanger Allee 101, Jena, 07743, Germany; 3 Lilly Research Laboratories, Eli Lilly and Co., Lilly Corporate Center, Indianapolis, Indiana, 46285, USA; 4 i3 StatProbe, 1001 Winstead Drive, Cary, North Carolina, 27513, USA; 5 Intensive Care Unit, Guy's and St Thomas' Hospital, Lambeth Palace Road, London, SE1 7EH, UK

## Abstract

**Introduction:**

Since the launch of drotrecogin alfa activated (DrotAA), institutions and individual countries have published data on its use in clinical practice, based on audit or registry data. These studies were limited in size and geographic locale and included patients with greater disease severity and higher mortality than those in clinical trials. The purpose of this study was to compare baseline characteristics and clinical outcomes (using appropriate statistical adjustments) of patients treated or not treated with DrotAA from the international PROGRESS (Promoting Global Research Excellence in Severe Sepsis) cohort study of severe sepsis.

**Methods:**

PROGRESS was a global, non-interventional, multi-center, prospective, observational study of patients having a diagnosis of severe sepsis treated in intensive care units at a participating institution. All treatment modalities were as per standard of care at the participating institutions. Baseline characteristics and hospital mortality were analyzed and regression techniques used to develop propensity and outcome models adjusted for baseline imbalances between groups.

**Results:**

Overall, 14,543 patients from 37 countries were enrolled and 12,492 had complete data for analysis. Germany was the highest enrolling country (1,810; 14.5%) and the US had the most DrotAA patients (206, 23.3%); 882 (7%) overall received DrotAA therapy. DrotAA-treated patients were younger (median age 58 vs. 61 years), had greater organ dysfunction (cardiovascular: 90% vs. 74%; respiratory: 90% vs. 81%; renal: 60% vs. 45%; metabolic: 63% versus 42%; 3 or more organ dysfunctions: 84% vs. 67%) and had a higher median APACHE II score (26 vs. 23, all with *P *< 0.001). Although in-hospital mortality was similar for DrotAA and non-DrotAA-treated patients (49.6% vs. 49.7%, respectively), after adjusting for imbalances, patients receiving DrotAA had a 28% (0.60 to 0.86, 95% Confidence Intervals) reduction in the odds of death and a relative risk reduction of 17% (*P *= 0.0003).

**Conclusions:**

In the PROGRESS registry, DrotAA-treated patients were younger, more severely ill, and had fewer co-morbidities than patients not treated with DrotAA. After adjustment for group differences, a significant reduction in the odds of death was observed for patients that received DrotAA compared with those that did not.

## Introduction

Although sepsis is the most common cause of mortality in noncoronary intensive care units (ICU) and the 10th leading cause of death overall in the US, few therapies have improved survival in large clinical trials [1-4]. Drotrecogin alfa (activated) (DrotAA; recombinant human protein C) has been approved for the treatment of adult patients with severe sepsis and multiple organ failure when added to the best standard care in Europe, and for treatment of those at high risk of death, for example, with Acute Physiology and Chronic Health Evaluation II (APACHE II) scores of 25 or more, in the US.

DrotAA was approved on the basis of a significant mortality reduction observed in the phase 3 Recombinant Human Activated Protein C Worldwide Evaluation in Severe Sepsis (PROWESS) clinical trial [5], together with supportive evidence from a phase 2 study [6]. As with any drug, it is pertinent to ask how the efficacy demonstrated within the confines of a clinical trial translates into effectiveness in every day clinical practice. Since its approval, a number of institutions and countries have published data on the use of DrotAA in clinical practice, based on audit or registry information; with the intent of defining 'real world' experience [7-15].

Disease severity and mortality rates tend to be higher in registries or databases than in clinical trials. In nonrandomized studies designed to examine the effectiveness of DrotAA, differences in geography, sample size, data collected, completeness of data collected, comparator groups, and statistical assessment have all varied, making it difficult to directly compare studies. A multi-center Canadian study [9] suggested early DrotAA administration was associated with lower mortality and noted higher bleeding rates compared with the PROWESS study. The Polish Registry [10] reported lower mortality in DrotAA-treated compared with non-treated patients and multivariate logistic regression modeling indicated that DrotAA use was the most significant factor reducing mortality in severe sepsis, irrespective of age and clinical condition. An Italian national survey suggested increased bleeding compared with the PROWESS study and significantly reduced crude ICU mortality compared to controls [7]. However, multivariate analysis suggested DrotAA treatment was associated with higher mortality after scheduled surgery. Analyses from a UK audit suggested that a mortality reduction observed in DrotAA-treated patients, compared with matched controls, was consistent with results from the PROWESS trial [12].

A retrospective review of ICU charts and medical records in the UK found that patients who had received DrotAA had a lower mortality rate than that predicted from APACHE II score and organ dysfunctions [11]. In another retrospective study of patients who had received DrotAA in the US, overall mortality was higher than in the PROWESS study, but patients were younger, had more comorbidities, had greater severity of illness and had a longer mean time from severe sepsis onset to the start of treatment with DrotAA. In patients treated within one day of severe sepsis onset, mortality was similar to those patients in the PROWESS trial with an APACHE II score of 25 or more [15]. PREMISS (Protocole en Réanimation d'Evaluation Médico économique d'une Innovation dans le Sepsis Sévère), a recent, prospective, observational, French study that assessed patients recruited before and after DrotAA licensing, reported that in matched samples from 'real life clinical practice' there was a 75% chance that DrotAA would be cost effective, depending on the 'willingness to pay threshold' [8]. Mortality of the DrotAA group after licensing was numerically reduced by 3.3%.

Finally, Belgian Reimbursement Registry results suggested DrotAA treatment was associated with a mortality reduction compared with Belgian patients not treated with DrotAA from the Promoting Global Research Excellence in Severe Sepsis (PROGRESS) database, after appropriate statistical adjustments for baseline differences [14]. It is important to note that the PROGRESS patients used in these analyses were not treated with DrotAA. Similar results were observed from an individual Belgian hospital using similar techniques and comparisons to Belgian Reimbursement Registry data and the PROWESS trial [13]. Although these studies have largely supported a beneficial effect of DrotAA, after adjusting for imbalances, debate continues as to how well the clinical trial mortality reduction associated with DrotAA treatment translates into real world clinical benefit [16,17].

The global PROGRESS registry was developed and designed with the intention of documenting profiles of disease diagnosis (epidemiologic, etiologic, and baseline disease severity data), patient management, and outcomes in real-life clinical settings across several regions of the world. The initial results from this database have recently been reported [18]. Although the PROGRESS registry was not specifically designed to assess the use of DrotAA, it was one of a number of therapeutic interventions on which data was collected. The purpose of the study presented here is to compare baseline characteristics and outcomes of patients receiving DrotAA with those not receiving DrotAA from an appropriately powered international cohort, using appropriate statistical adjustments.

## Materials and methods

### Study design

PROGRESS was an international, non-interventional, multi-center, prospective, observational study of patients with severe sepsis treated in ICUs. The study was supported by Eli Lilly and Company and executed with the oversight of a steering committee with clear governance rules covering data access, ownership, and publication. This publication was approved by the advisory committee. Patients entered into the study were treated as per the local standard of care without study-specific interventions. Evaluations, procedures, or treatment beyond those used as part of each institution's standard of care were not performed. As a result, ethical review board approval and informed consent were not a uniform requirement; however, most countries obtained ethics review board approval to confirm that informed consent was not required.

### Patients

Patients entered into the study must have had a diagnosis of severe sepsis (i.e. two or more systemic inflammatory response syndrome (SIRS) criteria, evidence of infection, and at least one sepsis-induced organ dysfunction (OD)). The definition of severe sepsis used in PROGRESS has been previously described [19] and has undergone recent updates and wider acceptance [20]. Although there was no age limit for participation in the study, this manuscript reports on patients aged 18 years and over enrolled in the registry.

### Data collection

The methods of data collection have been described in detail elsewhere [18]. Briefly, for each patient entered into the study, the participating physician or other investigative site personnel completed an electronic data form via a dedicated, secure website. When data entry was completed (i.e. baseline measurements, therapies, follow-up, outcome etc.), the patient record was closed by investigative site personnel. Patients with records that remained incomplete due to data (n = 258) or technical limitations (n = 130) were not included in the reporting database. Due to the nature of the database, safety information was not captured.

### Statistical methods

The primary objective of this study was to document demographics, management, and outcomes in patients with severe sepsis across several regions of the world. Baseline characteristics and hospital mortality of patients who did and did not receive DrotAA were analyzed. Continuous variables were summarized as means and analyzed using nonparametric analysis of variance (ANOVA), which is equivalent to the Wilcoxon rank-sum test in the two-group case. Qualitative variables were summarized with frequencies and analysed using Pearson chi-squared test.

Due to the nonrandomized nature of the study, baseline imbalances between patients with and without DrotAA therapy existed, therefore an adjusted mortality analysis was performed using propensity scores [21]. Additional details and discussion concerning propensity score development can be viewed in Additional data file 1. Variables used as candidates in the modeling process were clinically relevant baseline variables for which relatively few data values were missing and were strong univariate predictors of the response variable. The response variable for the propensity model was the dichotomous variable for treatment, DrotAA vs. non-DrotAA. The response variable for the mortality model was the dichotomous variable for hospital mortality, alive vs. dead. Most variables were analyzed using the values that were actually collected, but the levels of some multinomial variables (e.g. region) were collapsed to facilitate the construction of stable models.

Model development was broken into two phases: propensity model development and mortality model development. The propensity model consisted of stepwise elimination procedures and assessment of improvement in baseline imbalances [Table S1 in Additional data file 1 for additional details]. Mortality models consisted of similar stepwise elimination procedures and incorporation of results from the propensity model.

Both models were developed using a variant of backwards stepwise logistic regression. Model development began with inclusion of more than 45 candidate variables. Variables were evaluated and those with the highest *P *value (i.e. non-significant) sequentially eliminated in subsequent rounds of analysis until all variables in the model were statistically significant as determined by the Wald chi-squared test and a pre-specified exit threshold of 0.05 (e.g. model 17 in results section, with no further development).

Once all variables were significant, elimination was suspended, and those previously eliminated were reevaluated individually and reintroduced into the model one at a time. If the variable was found to be statistically significant (i.e. ≤ 0.05) in the current model setting, it was left in. If adding this variable to the model resulted in a different variable becoming insignificant, the insignificant variable was dropped in the next round of development. This process was continued until all variables in the model were statistically and clinically relevant (e.g. models 6–12, and 17 in results section).

Additional mortality models were developed in a forward manner. These models began with one covariate, propensity quartiles, and tested addition and exclusion of variables representative of DrotAA use (e.g. models 1 to 5 in results section). Additional mortality models were developed to test specific combinations of covariates (e.g. model 16 in results section). Several models were developed to illustrate the effect of including raw and imputed APACHE II score in multivariate models (e.g. models 13 to 15 in results section). Model performance was determined by the R-square and Hosmer and Lemeshow Goodness of Fit statistics. Odds ratios for the mortality model along with their 95% confidence intervals were also calculated for treatment vs. no treatment. Estimates of relative risk reduction were based on the average patient using the formula presented by Zhang and Yu [22].

The final propensity model developed to determine treatment administration had 12 variables: Age, Central Nervous System Failure, Metabolic Abnormalities, Hepatic Organ Failure, Renal Organ Failure, Hematologic Organ Failure, Respiratory Organ Failure, Cardiovascular Organ Failure, Vital Support-Vasopressors, Vital Support-Low Molecular Weight Heparin, Sepsis Treatment-Low Dose Steroids, and Chronic Renal Insufficiency.

Country was not used as a factor to predict either treatment administration or mortality because of low counts and outside influences (e.g. availability of DrotAA, methods of payment, reimbursement programs, treatment philosophies that inconsistently affected DrotAA usage across countries and could not be adjusted for in the model). APACHE II score was not used in the propensity model, due to the large number of missing observations (about 27%). However, mortality models with the APACHE II score both raw and imputed are presented for comparative purposes only.

Active Cancer is the only comorbidity variable presented in the mortality model table due to low data counts, lack of predictability, or multivariate parameter estimates with biologically implausible behavior in the multivariate setting based on clinical knowledge of the other variables representing comorbidity.

## Results

**Figure 1:**
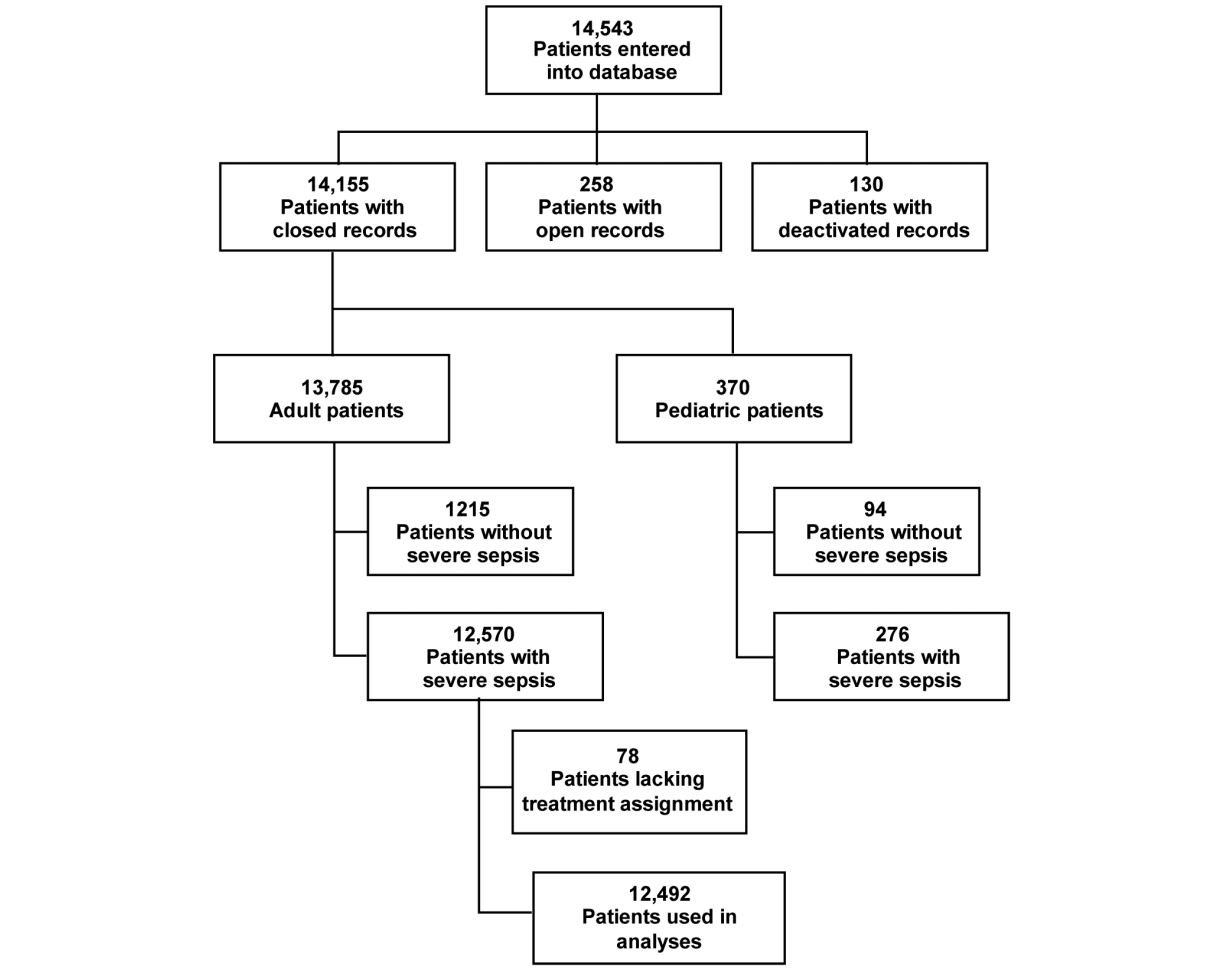
Patients were enrolled from December 2002 until December 2005 in 37 countries at 289 sites. There were 14,543 patients entered into the database of which 12,492 could be used for analyses.

PROGRESS was an observational study performed in 37 countries at 289 sites. The PROGRESS website enrolled patients from December 2002 until December 2005. Patient eligibility and enrollment is shown in Figure 1. There were 14,543 patients entered in the PROGRESS database. Of these, 388 had records that could not be verified and closed and 370 were pediatric patients. In 1215 of the resulting 13,785 patients, sites were unable to confirm a final diagnosis of severe sepsis. Of the 12,570 patients with closed records and confirmed severe sepsis, 78 had missing DrotAA treatment assignment, leading to 12,492 patients being used in these analyses [18].

Demographics of adult patients with severe sepsis from the PROGRESS registry are displayed in Table 1. Approximately 7% of the patients in the database received DrotAA (882 of 12,492). There were significant imbalances between patients that received DrotAA and those that did not in all baseline demographics except for gender. Patients were more likely to have been administered DrotAA (i.e. greater than the overall rate of 7%) if they were younger, or if they were from the US. Patients were also more likely to have received DrotAA if they were transferred from the emergency room (ER; 8.6%, 238 of 2760), another hospital (8.6%, 143 of 1658), or another ICU (12.2%, 47 of 385).

**Table 1:** PROGRESS demographics

**Variable**	**DrotAA** **n = 882**	**Non-DrotAA** **n = 11,610**	**Total** **n = 12,492**	***P *value**
Gender (male), n (%)	511 (57.9)	6903 (59.5)	7414 (59.3)	0.38
Age, years	57.6 (± 16.8)	60.6 (± 17.5)	60.3 (± 17.5)	<0.001
**Ethnicity, n (%):**	(n = 777)	(n = 10,247)	(n = 11,204)	<0.001
Caucasian	479 (61.6)	4933 (47.3)	5412 (48.3)	
Hispanic	127 (16.3)	2353 (22.6)	2480 (22.1)	
East/South East Asia	55 (7.1)	2091 (20.1)	2146 (19.2)	
Other	48 (6.2)	283 (2.7)	331 (3.0)	
African descent	40 (5.1)	194 (1.9)	234 (2.1)	
Western Asia	28 (3.6)	573 (5.5)	601 (5.4)	
**Region, n (%):**	(n = 882)	(n = 11,610)	(n = 12,492)	<0.001
US/Canada	307 (34.8)	1666 (14.3)	1973 (15.8)	
European Union	187 (21.2)	2492 (21.5)	2679 (21.4)	
Other	388 (44.0)	7452 (64.2)	7840 (62.8)	
**Referral pattern, n (%):**	(n = 876)	(n = 11,535)	(n = 12,410)	<0.001
ICU transfer from ER	238 (27.2)	2522 (21.9)	2760 (22.2)	
ICU transfer from ward	206 (23.5)	3214 (27.9)	3420 (27.6)	
ICU transfer from OR	156 (17.8)	2408 (20.9)	2564 (20.7)	
ICU transfer from other hospital	143 (16.3)	1515 (13.1)	1658 (13.4)	
ICU transfer from other ICU	47 (5.4)	338 (2.9)	385 (3.1)	
Direct to ICU from community	40 (4.6)	790 (6.8)	830 (6.7)	
Direct to ICU from intermediate care	40 (4.6)	654 (5.7)	694 (5.6)	
ICU transfer from another chronic care facility	4 (0.5)	51 (0.4)	55 (0.4)	
Other/unknown	2 (0.2)	42 (0.4)	44 (0.4)	

DrotAA = drotrecogin alfa (activated); ER = emergency room; ICU = intensive care unit; OR = operating room; PROGRESS = Promoting Global Research Excellence in Severe Sepsis.

Table 2 presents clinical characteristics of PROGRESS adult patients with severe sepsis. In all clinical characteristics, with the exception of patients with Gram-negative infections and the primary site of infection, baseline imbalances were present between patients who received DrotAA and those that did not. Elective surgical patients (surgery performed in the previous seven days but scheduled more than 24 hours prior to ICU admission) were more likely to have received DrotAA (9.5%, 127 of 1332) than patients who had emergency surgery (6.3%, 215 of 3388). A greater proportion of patients with four SIRS criteria received DrotAA (8.4%, 526 of 6289), as well as those with community acquired infections (8.1%, 529 of 6562). Patients with fungal infections were more likely to be treated with DrotAA (9.5%, 103 of 1089), followed closely by Gram-positive infections (9.2%, 371 of 4039).

**Table 2:** PROGRESS clinical characteristics

**Variable**	**DrotAA** **n = 882**	**Non-DrotAA** **n = 11,610**	**Total** **n = 12,492**	***P *value**
**Medical/Surgical Status, n (%):**				<0.001
Medical	540 (61.2)	7232 (62.3)	7772 (62.2)	
Surgical – Elective	127 (14.4)	1205 (10.4)	1332 (10.7)	
Surgical – Emergency	215 (24.4)	3173 (27.3)	3388 (27.1)	
**SIRS criteria met, n (%):**	(n = 858)	(n = 11,241)	(n = 12099)	<0.001
0	2 (0.2)	29 (0.3)	31 (0.3)	
1	5 (0.6)	188 (1.7)	193 (1.6)	
2	57 (6.6)	1198 (10.7)	1255 (10.4)	
3	268 (31.2)	4063 (36.1)	4331 (35.8)	
4	526 (61.3)	5763 (51.3)	6289 (52.0)	
**Source of infection, n (%):**	(n = 856)	(n = 11,260)	(n = 12,116)	<0.001
Community acquired	529 (61.8)	6033 (53.6)	6562 (54.2)	
Nosocomial – hospital acquired	217 (25.4)	3172 (28.2)	3389 (28.0)	
Nosocomial – ICU acquired	110 (12.9)	2055 (18.3)	2165 (17.9)	
**Type of infection, n (%):**	(n = 675)	(n = 8380)	(n = 9055)	
Gram negative	392 (58.1)	4795 (57.2)	5187 (57.3)	0.67
	(n = 681)	(n = 8396)	(n = 9077)	
Gram positive	371 (54.5)	3668 (43.7)	4039 (44.5)	<0.001
	(n = 682)	(n = 8678)	(n = 9360)	
Fungal	103 (15.1)	986 (11.4)	1089 (11.6)	0.003
**Primary site of infection, n (%):**	(n = 861)	(n = 11,301)	(n = 12,162)	0.35
Abdominopelvic	227 (26.4)	2642 (23.4)	2869 (23.6)	
Bone or joint	13 (1.5)	160 (1.4)	173 (1.4)	
Hematogenous	59 (6.9)	738 (6.5)	797 (6.6)	
Indwelling catheter-dialysis access	3 (0.3)	79 (0.7)	82 (0.7)	
Indwelling catheter-vascular access	11 (1.3)	163 (1.4)	174 (1.4)	
Lung	366 (42.5)	5282 (46.7)	5648 (46.4)	
Meninges	12 (1.4)	169 (1.5)	181 (1.5)	
Skin or skin structure	44 (5.1)	586 (5.2)	630 (5.2)	
Urinary tract	71 (8.2)	887 (7.8)	958 (7.9)	
Other	55 (6.4)	595 (5.3)	650 (5.3)	

DrotAA = drotrecogin alfa (activated); ICU = intensive care unit; PROGRESS = Promoting Global Research Excellence in Severe Sepsis; SIRS = Systemic Inflammatory Response Syndrome.

Intensive care treatment that patients in the PROGRESS database received is displayed in Table 3. In all instances except when receiving unfractionated heparin, patients administered DrotAA received significantly higher levels of care. Particularly notable (>15% difference) were the higher levels of vasopressor use, low-dose steroid use, and use of mechanical prophylaxis for venous thrombotic events (VTE).

**Table 3:** PROGRESS intensive care treatment

**Variable**	**DrotAA** **n = 882**	**Non-DrotAA** **n = 11,610**	**Total** **n = 12,492**	***P *value**
IV fluid resuscitation, n (%)	(n = 782) 718 (91.8)	(n = 10,492) 9053 (86.3)	(n = 11,274) 9771 (86.7)	<0.001
Mechanical ventilation, n (%)	(n = 882) 842 (95.5)	(n = 11,609) 9838 (84.7)	(n = 12,491) 10,680 (85.5)	<0.001
Vasopressors, n (%)	(n = 882) 828 (93.9)	(n = 11,608) 9005 (77.6)	(n = 12,490) 9833 (78.7)	<0.001
**Nutrition:**				
Enteral, n (%)	(n = 882) 678 (76.9)	(n = 11,588) 8369 (72.2)	(n = 12,470) 9047 (72.6)	0.003
Parenteral, n (%)	(n = 882) 383 (43.4)	(n = 11,601) 3726 (32.1)	(n = 12,483) 4109 (32.9)	<0.001
**Heparin:**				
Low molecular weight	(n = 871) 418 (48.0)	(n = 11,586) 3879 (33.5)	(n = 12,457) 4297 (34.5)	<0.001
Unfractionated	(n = 868) 345 (39.8)	(n = 11,601) 4628 (39.9)	(n = 12,469) 4973 (39.9)	0.932
**Steroids:**				
Low dose	(n = 878) 499 (56.8)	(n = 11,559) 3994 (34.6)	(n = 12,437) 4493 (36.1)	<0.001
High dose	(n = 880) 156 (17.7)	(n = 11,600) 1397 (12.0)	(n = 12,480) 1553 (12.4)	<0.001
Mechanical VTE prophylaxis	(n = 765) 295 (38.6)	(n = 10,371) 2407 (23.2)	(n = 11,136) 2702 (24.3)	<0.001
Renal replacement therapy	(n = 876) 283 (32.3)	(n = 11,598) 2383 (20.6)	(n = 12,474) 2666 (21.4)	<0.001
Platelet transfusion	(n = 780) 178 (22.8)	(n = 10,483) 1677 (16.0)	(n = 11,263) 1855 (16.5)	<0.001

DrotAA = drotrecogin alfa (activated); PROGRESS = Promoting Global Research Excellence in Severe Sepsis; VTE = venous thromboembolism.

Disease severity measures of patients in the database are presented in Table 4. Although DrotAA patients were younger (Table 1), they had statistically significant higher disease severity scores by all measures except for Simplified Acute Physiology Score II (SAPS II) score (the smallest subgroup). Consistent with this, a greater proportion of patients receiving DrotAA had three or more OD and DrotAA patients also experienced a greater degree of cardiovascular, respiratory, renal, and metabolic OD.

**Table 4:** PROGRESS disease severity

**Variable**	**DrotAA** **n = 882**	**Non-DrotAA** **n = 11,610**	**Total** **n = 12,492**	***P *value**
**Number of organ dysfunctions (OD):**	(n = 703)	(n = 8910)	(n = 9613)	<0.001
1	18 (2.6)	1042 (11.7)	1060 (11.0)	
2	93 (13.2)	1861 (20.9)	1954 (20.3)	
3	169 (24.0)	2045 (23.0)	2214 (23.0)	
4	161 (22.9)	1778 (20.0)	1939 (20.2)	
5	143 (20.3)	1171 (13.1)	1314 (13.7)	
6	87 (12.4)	677 (7.6)	764 (8.0)	
7	32 (4.6)	336 (3.8)	368 (3.8)	
**Type of dysfunction, n (%):**				
Cardiovascular	(n = 882) 791 (89.7)	(n = 11,565) 8536 (73.8)	(n = 12,447) 9327 (74.9)	<0.001
Respiratory	(n = 880) 788 (89.5)	(n = 11,550) 9357 (81.0)	(n = 12,430) 10,145 (81.6)	<0.001
Hematologic	(n = 879) 314 (35.7)	(n = 11,475) 3773 (32.9)	(n = 12,354) 4087 (33.1)	0.08
Renal	(n = 879) 523 (59.5)	(n = 11,480) 5117 (44.6)	(n = 12,359) 5640 (45.6)	<0.001
Hepatic	(n = 771) 150 (19.5)	(n = 10,188) 2111 (20.7)	(n = 10,959) 2261 (20.6)	0.40
Metabolic	(n = 867) 542 (62.5)	(n = 11,266) 4745 (42.1)	(n = 12,133) 5287 (43.6)	<0.001
Central nervous system	(n = 767) 276 (36.0)	(n = 9987) 3686 (36.9)	(n = 10,754) 3962 (36.8)	0.61
APACHE II score, mean (± SD)	(n = 610) 25.6 (± 8.5)	(n = 8513) 23.2 (± 8.2)	(n = 9123) 23.4 (± 8.3)	<0.001
Total SOFA score, mean (± SD)	(n = 334) 10.3 (± 3.4)	(n = 4785) 9.2 (± 3.9)	(n = 5119) 9.3 (± 3.9)	<0.001
Total MODS score, mean (± SD)	(n = 171) 8.6 (± 3.7)	(n = 2244) 6.4 (± 3.6)	(n = 2415) 6.5 (± 3.6)	<0.001
SAPS II score, mean (± SD)	(n = 165) 50.2 (± 18.5)	(n = 2831) 49.0 (± 17.3)	(n = 2996) 49.1 (± 17.4)	0.48

APACHE II = Acute Physiology and Chronic Health Evaluation II; DrotAA = drotrecogin alfa (activated); MODS = multiple organ dysfunction syndrome; PROGRESS = Promoting Global Research Excellence in Severe Sepsis; SAPS II = Simplified Acute Physiology Score II; SD = standard deviation; SOFA = Sequential Organ Failure Assessment.

Table 5 displays the comorbidities of patients in PROGRESS. Departing somewhat from previous parameters, particularly supportive care and disease severity, there were proportionately fewer imbalances, and comorbidities were all numerically greater (as measured by percentage) in the non-DrotAA group. Patients receiving DrotAA treatment were significantly less likely to have active cancer, congestive heart failure, and chronic renal insufficiency compared with those who did not receive DrotAA.

**Table 5:** PROGRESS comorbidities

**Variable**	**DrotAA** **n = 882**	**Non-DrotAA** **n = 11,610**	**Total** **n = 12,492**	***P *value**
Diabetes, n (%)	(n = 766) 164 (21.4)	(n = 10,352) 2441 (23.6)	(n = 11,118) 2605 (23.4)	0.17
Chronic lung disease, n (%)	(n = 870) 136 (15.6)	(n = 11,447) 1924 (16.8)	(n = 12,317) 2060 (16.7)	0.37
Active cancer, n (%)	(n = 844) 102 (12.1)	(n = 11,101) 1787 (16.1)	(n = 11,945) 1889 (15.8)	0.002
Congestive heart failure, n (%)	(n = 879) 97 (11.0)	(n = 11,460) 1630 (14.2)	(n = 12,339) 1727 (14.0)	0.009
Chronic renal insufficiency, n (%)	(n = 872) 54 (6.2)	(n = 11,481) 1285 (11.2)	(n = 12,353) 1339 (10.8)	<0.001
Chronic liver disease, n (%)	(n = 843) 48 (5.7)	(n = 11,142) 727 (6.5)	(n = 11,985) 775 (6.5)	0.34
Other, n (%)	(n = 768) 197 (25.7)	(n = 10,369) 2473 (23.9)	(n = 11,125) 2670 (24.0)	0.90

DrotAA = drotrecogin alfa (activated); PROGRESS = Promoting Global Research Excellence in Severe Sepsis.

Country-specific enrollment and DrotAA use data are presented in Table 6. Germany was the highest enrolling country of the PROGRESS Registry and the US had twice the number of patients treated with DrotAA as the next highest country (Canada). Enrollment numbers did not correlate with DrotAA use.

**Table 6:** PROGRESS 10 highest enrolling countries and 10 highest drotrecogin alfa (activated) using countries

			**DrotAA-treated patients**	
			
**Country**	**Number of sites**	**Enrolled patients,** **n (% overall)** **(n = 12,492)**	**n, (% of total DrotAA patients)** **[Overall rank]** **(n = 882)**	**Within-country DrotAA use,** **% (DrotAA patients/Total patients) [Rank]**
Germany*	**17**	1810 (14.5)	98 (11.1) [3]	5.4 (98/1810) [11]
Argentina*	**18**	1269 (10.1)	22 (2.5) [9]	1.7 (22/1269) [15]
Canada*^†^	**12**	1213 (9.7)	101 (11.5) [2]	8.3 (101/1213) [7]
Brazil*^†^	**9**	968 (7.7)	65 (7.4) [4]	6.7 (65/968) [9]
India*	**21**	803 (6.4)	29 (3.3) [8]	3.6 (29/803) [12]
United States*^†^	**26**	760 (6.1)	206 (23.3) [1]	27.1 (206/760) [2]
Australia*^†^	**4**	667 (5.3)	53 (6.0) [6]	7.9 (53/667) [8]
Malaysia*	**4**	641 (5.1)	12 (1.4) [11]	1.9 (12/641) [14]
Philippines*	**10**	489 (3.9)	10 (1.1) [12]	2.0 (10/489) [13]
Mexico*^†^	**10**	475 (3.8)	54 (6.1) [5]	11.4 (54/475) [6]
Belgium^†^	**7**	360 (2.9)	43 (4.9) [7]	11.9 (43/360) [5]
Poland^†^	**10**	210 (1.7)	29 (3.3) [8]	13.8 (29/210) [4]
New Zealand^†^	**1**	145 (1.2)	9 (1.0) [13]	6.2 (9/145) [10]
Turkey^†^	**16**	128 (1.2)	43 (4.9) [7]	33.6 (43/128) [1]
Algeria^†^	**6**	105 (0.8)	19 (2.2) [10]	18.1 (19/105) [3]

* Top 10 enrolling countries.

^† ^Top 10 countries with highest within-country DrotAA use (enrollment >100 patients)

Numbers in brackets, [], relate to a country's rank for DrotAA patients and within country use

DrotAA = drotrecogin alfa (activated); MODS = multiple organ dysfunction syndrome; PROGRESS = Promoting Global Research Excellence in Severe Sepsis.

The effect of treatment with DrotAA on in-hospital mortality was assessed by developing a collection of logistic regression models. Multiple models were developed to ensure that conclusions of the analysis were not being dominated by individual covariates and to present variations of differing models with statistically relevant and clinically logical covariates. A suite of models is presented in Table 7 to represent consistency of results across varying methodologies and covariate combinations. Each model was comprised of differing covariates and combinations of covariates and is presented with the corresponding performance statistics, odds ratios, and relative risk reduction calculations for treatment. Other than the first two models, presented only as baseline representation, the R^2 ^values for each model were relatively similar, ranging from 0.128 to 0.278. Model fit did vary depending on the covariate combinations, but overall relative risk reduction, ranging from 13% to 18%, shows that regardless of the choice of statistically significant and clinically relevant covariates or combinations of covariates, treatment with DrotAA consistently resulted in a relative reduction in the risk of mortality. Given that the US contributed the largest proportion of DrotAA patients for a single country, sensitivity analyses were run without the US data, and similar results were observed (i.e. all double digit relative risk reduction, data not shown).

**Table 7:** Summary of multilvariate logistic regression model development

**Model**	**Hospital Mortality Adjusted by Treatment and**	**n**	**Adjusted R^2 ^Value**	**Goodness of Fit Chi-square**	***P *value for Treatment Factor in Multivariate Model**	**Odds Ratio,** Point Estimate (95% CI*)	**Relative Risk Reduction**
1	Propensity Quartiles^†^	8806	0.034	0.002	0.0005	0.745 (0.631, 0.879)	15%
2	Age, Propensity Quartiles	8806	0.083	0.548	0.001	0.755 (0.638, 0.893)	14%
3	Age, 7 OD^1^	8939	0.159	0.073	0.0056	0.788 (0.665, 0.933)	13%
4	7 OD, Propensity Quartiles	8806	0.133	0.14	0.0002	0.722 (0.609, 0.857)	17%
5	Age, 7 OD, Propensity Quartiles	8806	0.161	0.258	0.0005	0.735 (0.618, 0.874)	16%
6	Age, 7 OD, Vasopressors, Propensity Quartiles	8806	0.190	0.178	0.0006	0.739 (0.622, 0.878)	16%
7	Age, 7 OD, Site of Infection, Propensity Quartiles	8806	0.173	0.68	0.0009	0.744 (0.625, 0.866)	15%
8	Age, 7 OD, Active Cancer Description, Propensity Quartiles	8806	0.170	0.821	0.0012	0.751 (0.631, 0.893)	15%
9	Age, 7 OD, Active Cancer, Propensity Quartiles	8408	0.167	0.952	0.0003	0.72 (0.603, 0.860)	17%
10	Age, 7 OD, Active Cancer, Source of Infection, Propensity Quartiles	8142	0.172	0.852	0.0002	0.712 (0.594, 0.854)	18%
11	Age, 7 OD, Active Cancer, Source of Infection, Vasopressors, Propensity Quartile	8142	0.200	0.131	0.0003	0.717 (0.598, 0.860)	18%
12	Age, 7 OD, Active Cancer, Vasopressors, Propensity Quartile	8408	0.197	0.278	0.0004	0.726 (0.607, 0.867)	18%
13	APACHE^2 ^II Score, Propensity Quartiles	6431	0.162	0.658	0.0007	0.692 (0.559, 0.856)	18%
14	Imputed APACHE II Score, Propensity Quartiles	8806	0.128	0.956	<0.0001	0.71 (0.599, 0.843)	17%
15	Age, 7 OD, Imputed APACHE II Score, Propensity Quartiles	8806	0.198	0.765	0.0002	0.714 (0.599, 0.851)	18%
16	Age, 7 OD, Region^3^, Propensity Quartiles	8806	0.170	0.559	0.004	0.77 (0.644, 0.92)	14%
17	Age, 4 OD (no Cardiologic, Metabolic, Renal), Mechanical Ventilation, Renal Replacement Therapy, Platelet Transfusion, Enteral Nutrition, Mechanical-VTE-Prophylaxis, LMWH, Active Cancer, Propensity Quartiles	8283	0.278	0.028	0.0115	0.783 (0.647, 0.947)	13%

* 95% Confidence Intervals (CI).

^† ^See Additional data file 1 for additional detail of propensity score methodology. Propensity quartiles represent subgroups in which the probability of receiving drotrecogin alfa (activated) was roughly constant. Imbalances in unobserved variables have not been accounted for as they would be in a randomized trial.

^1 ^Organ Dysfunctions (OD).

^2 ^Acute Physiology And Chronic Health Evaluation. APACHE II scores were missing for over 3300 patients. In models that include APACHE II score imputed, these missing values were imputed with the overall mean APACHE II score.

^3 ^Active Cancer Description had several categories (e.g. hematologic, solid tumor etc.), while Active Cancer was categorical (i.e. Yes/No).

^4 ^Region represents Europe, US/Canada, and Other.

LMWH = low molecular weight heparin; VTE = venous thromboembolism.

Overall registry mortality remained stable over the duration of the study (data not shown). Clinical outcomes and mortality data are presented in Table 8. Numerically fewer DrotAA patients were discharged to the community and more were discharged to extended or chronic care institutions, compared with those patients not receiving DrotAA. Although in-hospital mortality was similar between groups, after adjusting for imbalances (see above), patients receiving DrotAA had a 28% reduction in the odds of death and a relative risk reduction of 17% (using Model 9). Model covariates were age, separate flags for seven ODs (cardiovascular, respiratory, hematologic, renal, hepatic, metabolic, and central nervous system), active cancer, and propensity quartile.

**Table 8:** PROGRESS DrotAA mortality and clinical outcomes

**Variable**	**DrotAA** **n = 882**	**Non-DrotAA** **n = 11,610**	**Total** **n = 12,492**	***P *value**
**Discharge location by DrotAA use, n (%)**:	(n = 807)	(n = 10,537)	(n = 11,344)	<0.001
Died	400 (49.6)	5236 (49.7)	5636 (49.7)	
Community	236 (29.2)	3652 (34.7)	3888 (34.3)	
Other hospital	73 (9.0)	856 (8.1)	929 (8.2)	
Extended/Chronic care Institution	83 (10.3)	620 (5.9)	703 (6.2)	
Other/Unknown	15 (1.9)	173 (1.6)	188 (1.7)	
Hospital mortality for DrotAA therapy, adjusted for Age, 7 OD*, Active Cancer, and Propensity Quartiles^†^	Adjusted Odds Ratio	95% Confidence Interval	----	*P *value
All patients**	0.72	0.603 – 0.86	----	0.0003

*Organ Dysfunction (OD).

^† ^See METHODS and Additional data file 1 for details and explanation of this covariate

** Model #9 used for this analysis.

DrotAA = drotrecogin alfa (activated); PROGRESS = Promoting Global Research Excellence in Severe Sepsis.

## Discussion

With 12,492 patients in 37 countries, PROGRESS is the largest severe sepsis registry to date. PROGRESS has provided important information on the use of DrotAA in everyday clinical practice, in addition to providing information on treatment variation across regions and countries. There were significant differences in most baseline characteristics between patients receiving DrotAA and those that did not. DrotAA-treated patients were younger, more severely ill and received higher levels of support but had fewer comorbidities. DrotAA-treated patients also had more Gram-positive and fungal infections, although the relevance of this, if any, is unclear, as microbiologic diagnosis would be anticipated to occur later than the decision to treat with DrotAA. Although in-hospital mortality was similar between groups, when adjusted for imbalances, DrotAA patients had significantly lower odds of death.

In comparing the PROGRESS severe sepsis registry with others, it should be noted that PROGRESS was an international registry. Previous analyses of DrotAA-treated patients have been registries for individual institutions or countries [7-15]. In all cases, including PROGRESS, there was higher disease severity and mortality in comparison to severe sepsis clinical trials. Mortality results for PROGRESS are broadly consistent with results of the other registries, which have generally reported a lower mortality with DrotAA treatment in either crude or adjusted mortality comparisons. This is important considering the different approaches used to address the lack of a randomized control group. Although the results presented for the PROGRESS registry do not resolve the risk/benefit controversy surrounding DrotAA treatment (i.e. no safety data collected), they do provide additional information from a much larger and diverse patient population that are consistent with previous data.

Although there were missing values for some variables, this occurrence was relatively uncommon and allowed for a more robust statistical analysis than would be possible with smaller cohorts, especially for subgroup analyses. In addition, the large number of centers and countries involved provided a more detailed and encompassing picture of how DrotAA is currently being prescribed in various countries around the world.

Following the publication of the surviving sepsis campaign (SSC) guidelines [23,24] a number of countries and hospitals have reported improved outcome in critically ill patients following the introduction of these evidence-based sepsis 'bundle' protocols [25-30]; however, due to the nature of such studies, it is not possible to determine the relative contributions of the different interventions. When considering how evolving practice and institution of the SSC recommended guidelines may have affected mortality of the PROGRESS study, it is important to note that the first set of SSC guidelines were published in March 2004, and that recruitment in PROGRESS in many countries was largely completed by the end of 2004. Therefore, the SSC guidelines existed for only a small portion of the study duration, and thus the PROGRESS database will have a limited ability to detect the full effects of these guidelines. In PROGRESS, although individual country mortality may have increased or decreased over the duration of the study, overall mortality remained unchanged (data not shown).

The emerging picture of the patient being treated with DrotAA in the PROGRESS study is very interesting. In PROGRESS, DrotAA-treated patients were younger, had higher disease severity, received greater levels of supportive care, yet had fewer comorbidities compared with those not treated with DrotAA. This is somewhat different from the picture of a slightly older patient with several comorbidities and slightly less disease severity that has generally come from randomized, placebo-controlled clinical trials in severe sepsis. The finding that DrotAA-treated patients were more often referred from other ICUs or hospitals and had more supportive/expensive care suggests they may have been treated late in the course of the disease, perhaps as a rescue therapy in the younger patient, after other therapies had failed. It may also imply that there is a developing practice to use DrotAA on those patients perceived as having the best opportunity to improve (i.e. youngest with most physiologic reserve and best long-term prognosis). It appears 'real world' physicians treat patients differently than physicians participating in a clinical trial. Low rates of DrotAA usage have been noted in other registries [12,31], which in part may be related to clinicians waiting until patients have higher disease severity, compared with clinical trials [15].

Referring to model development for mortality, given that data collection was not complete for all parameters, our intent was to use as simple a model as possible that provided rigorous, meaningful information. Many baseline imbalances were clinically meaningful, particularly age. Population quartiles, based on a propensity model, were developed to address these imbalances. Numerous mortality models were developed and presented. In general, the simplest models had lower performance statistics. Models with additional covariates increased the performance statistics only up to a point and then either the R^2 ^or goodness of fit statistic would suffer. The best models were those including age, seven ODs, and propensity quartiles in combination with additional statistically and clinically relevant covariates.

Country alone was not used as a covariate to predict either treatment administration or mortality primarily because of the wide variability in the number of patients that received DrotAA (i.e. approximately half of the countries had less than 10 patients that received DrotAA). However, there are likely to be other outside influences that inconsistently affected DrotAA usage across countries and could not be adjusted for in the model; for example, availability of DrotAA, methods of payment, reimbursement programs, and treatment philosophies of the country. APACHE II score was not used in the propensity model, due to the large number of missing observations. It was used for comparative purposes in the final mortality model. The most important conclusion from modeling was not the actual value presented for mortality reduction, rather that DrotAA treatment consistently reduced the risk of mortality regardless of the model used, when adjusted by data imbalances and demographic factors.

As with any observational study, there are inherent weaknesses with the PROGRESS study. The lack of a randomized control group clearly sets limitations to any inferences that might be drawn about the efficacy of DrotAA. A weakness of our approach to data collection was the reliance on local data entry without formal data monitoring against source records. Regarding site selection, sites within a country were not randomly selected. So it is possible that 'country' practice may not fully reflect the practice within that country, particularly for countries with relatively few sites. In addition, PROGRESS was not initiated in countries with well-established existing sepsis registries. Although PROGRESS involved large numbers of patients, there were still parameters with significant levels of missing measurements. This resulted in small patient numbers for certain characteristics and subgroups, which precluded rigorous statistical analyses. There were also characteristics not collected in the study, such as timing of OD and of various treatments received, which could have affected patient outcome. Relevant to this Wheeler and colleagues [15] recently reported a significant increase in hospital mortality rates in patients receiving DrotAA one or two or more calendar days after the diagnosis of severe sepsis, compared with those that received treatment on the day of diagnosis. Also relevant is that, not only were different centers and countries involved with different intra- and inter-country variability in standard of care, the various centers and countries were involved in the three-year study over different periods of time, which also could have affected the standard of care with evolving practice (e.g. SSC Guidelines [23,24]. Because PROGRESS was an observational study, it is difficult to explicitly determine to what extent the observed geographic variations in mortality resulted from the differences in baseline characteristics of the patients entered, differences in therapies patients received, genetic components, or other unrecorded factors. It should also be noted that the PROGRESS study was not prospectively designed to examine mortality by DrotAA use. Finally, due to the observational nature of the study, adverse events and safety events were not recorded and no risk/benefit analysis possible.

## Conclusions

The PROGRESS registry has helped document information on the use of DrotAA in everyday clinical practice and on treatment variation across regions and countries. DrotAA-treated patients were younger, more severely ill, received higher levels of support but had fewer comorbidities. Although safety information was not captured, when adjustments were made for imbalances, a significant reduction in the odds of death was observed for patients that received DrotAA compared with those that did not. These data are consistent with data from previous individual country registry data.

## Key messages

• The PROGRESS registry is one of the largest, if not the largest, severe sepsis registry to date.

• PROGRESS registry patients treated with DrotAA were younger, more severely ill, and had fewer comorbidities than patients not receiving it.

• PROGRESS patients treated with DrotAA had a significant reduction in the odds of death when appropriate adjustments were made.

• These data are consistent with data from previous individual country registry data.

## Abbreviations

ANOVA: analysis of variance; APACHE II: Acute Physiology and Chronic Health Evaluation II; DrotAA: drotrecogin alfa (activated); ER: emergency room; ICU: intensive care unit; OD: organ dysfunction; PREMISS: Protocole en Réanimation d'Evaluation Médico économique d'une Innovation dans le Sepsis Sévère; PROGRESS: Promoting Global Research Excellence in Severe Sepsis; PROWESS: Recombinant Human Activated Protein C Worldwide Evaluation in Severe Sepsis; SAPS II: Simplified Acute Physiology Score II; SIRS: Systemic Inflammatory Response Syndrome; SSC: Surviving Sepsis Campaign; VTE: venous thromboembolism.

## Competing interests

Drs Martin, Reinhart, and Beale have all served as consultants to and participated in Eli Lilly and Co sponsored trials. Dr Brunkhorst received research grants from Elli Lilly Deutschland GmbH. Dr Martin's institution received funding for Dr Martin conducting clinical trials with Eli Lilly and Co. Drs Janes and Sundin are employees and stockholders of Eli Lilly and Co. Ms. Garnett is a contractor for Eli Lilly and Co.

## Authors' contributions

RB, FB, KR, and JJ participated in the conception and design of the registry. RB, GM, FB, JJ, KG, and DPS contributed to the development and conduct of the principal analyses. All authors contributed to drafting and critically revising the manuscript and read and approved the final version of the manuscript.

## Supplementary Material

Additional file 1 A word file containing a more detailed methodology of the propensity model development and a table (Table S1 in Additional data file 1) demonstrating the balance among baseline characteristics between the two treatment groups achieved in the propensity quartiles. Click here for file
