# Supplementary material for: The international PROGRESS registry of patients with severe sepsis: drotrecogin alfa (activated) use and patient outcomes
Source: Crit Care. 2009 Jun 30;13(3):R103. doi: 10.1186/cc7936 (PMC2717475; doi:10.1186/cc7936)
Supplement: Additional file 1 — A word file containing a more detailed methodology of the propensity model development and a table (Table S1 in Additional data file 1) demonstrating the balance among baseline characteristics between the two treatment groups achieved in the propensity quartiles. [file cc7936-S1.docx]

**Additional data file 1**

Since the PROGRESS data were obtained from an observational rather than a randomized study, it was important to account for differences in baseline characteristics between the group of patients that was treated with DrotAA and the group that was not. The effect of imbalances between treatment groups was addressed using propensity scores. This approach enabled assessment of the comparability of baseline characteristics for the two treatment groups and facilitated straightforward estimation of treatment effects that incorporated adjustments for differences in patient characteristics at baseline.

It is important to note that propensity scores help to ameliorate imbalances across treatment groups only for those covariates that were measured in the study. Imbalances in unobserved variables have not been accounted for as they would be in a randomized trial. Patients in a randomized trial are assigned to treatment on a random basis, therefore all observed and unobserved characteristics of the patients can be considered to be balanced or consistent, and any differences in efficacy can be attributed to the treatment assignment. This approximate balance is not ensured in a nonrandomized study, where selection biases can occur. Propensity modeling offers an effective approach to adjust for these imbalances.

Propensity scores were derived by constructing multivariate models in which observed patient characteristics were used to predict the probability of receiving a particular treatment. Each patient in the study was assigned a propensity score that represented the estimated probability of receiving DrotAA. Patients were subclassified into quartiles on the basis of their propensity scores in order to achieve approximate balance in the treatment arms across all measured covariates (see Table S1 in Additional data file 1). These quartile assignments along with treatment and additional factors were used as covariates in the development of a series of logistic regression models with the dichotomous response of Mortality vs. non-Mortality.

The propensity model was constructed using stepwise logistic regression. Covariates found to be predictive of the therapy received were age, each of the seven types of ODs, chronic renal insufficiency, administration of vasopressors, administration of low molecular-weight heparin, and administration of low-dose corticosteroids.

As observed in Table S1 (in Additional data file 1), application of propensity scores led to a vast improvement in baseline balance across the two treatment groups in comparison to the imbalances that were present in the raw baseline data. The results also indicate that differences in the probability of receiving DrotAA were associated with differences in age, in the rates of cardiovascular, respiratory, renal, and metabolic dysfunction, and in the frequency of administration of vasopressors, low molecular-weight heparin, and low-dose corticosteroids

**Additional data file 1 Table S1: Improvement in Baseline Imbalance Using Propensity Score Analysis**

|  | | Quartile 1 | | | Quartile 2 | | | Quartile 3 | | | Quartile 4 | | |
| --- | --- | --- | --- | --- | --- | --- | --- | --- | --- | --- | --- | --- | --- |
|  | Original Baseline p-values | Non-DrotAA | DrotAA-treated | p | Non-DrotAA | DrotAA-treated | p | Non-DrotAA | DrotAA-treated | p | Non-DrotAA | DrotAA-treated | p |
|  |  | n=2323 | n=39 |  | n=2260 | n=104 |  | n=2210 | n=152 |  | n=1977 | n=386 |  |
| Age | <0.001 | 63 | 67 | 0.29 | 62 | 63 | 0.45 | 61 | 60 | 0.86 | 56 | 54 | 0.02 |
| Organ Dysfunctions:  Cardiovascular  Respiratory  Hematologic  Renal  Hepatic  Metabolic  Neurologic | <0.001  <0.001  0.08  <0.001  0.40  <0.001  0.61 | 38%  62  29  25  19  19  39 | 51%  62  33  33  26  28  33 | 0.10  0.92  0.54  0.23  0.29  0.15  0.51 | 77%  81  32  36  20  34  38 | 73%  87  29  33  20  36  38 | 0.72  0.15  0.48  0.48  0.96  0.68  0.94 | 92%  93  39  50  25  54  39 | 91%  93  38  50  22  49  32 | 0.01  0.82  0.84  0.98  0.43  0.31  0.08 | 97%  98  40  70  20  74  37 | 99%  98  41  74  19  79  39 | 0.03  0.65  0.79  0.08  0.78  0.05  0.24 |
| Vasopressors. | <0.001 | 42% | 51% | 0.24 | 84% | 90% | 0.07 | 96% | 93% | 0.20 | 99% | 99% | 0.89 |
| Chronic Renal Insufficiency | <0.001 | 19% | 15% | 0.60 | 15% | 14% | 0.96 | 8% | 9% | 0.95 | 3% | 3% | 0.59 |
| LMW Heparin | <0.001 | 18% | 10% | 0.19 | 28% | 27% | 0.80 | 39% | 41% | 0.60 | 61% | 67% | 0.02 |
| Low Dose Corticosteroids | <0.001 | 10% | 10% | 0.99 | 26% | 25% | 0.76 | 40% | 42% | 0.54 | 69% | 74% | 0.09 |
